# Shedding Light on an Invisible Population in Geriatric Medicine: Unmet Care Needs Among Homebound Older Adults

**DOI:** 10.1093/geroni/igaf122.1031

**Published:** 2025-12-31

**Authors:** Orla Sheehan, Caoimhe McManus, Katherine Ornstein, Benjamin Oseroff, Robert Zimbroff, Tiffany Riser

**Affiliations:** Royal College of Surgeons in Ireland, Dublin, Ireland; Connolly Hospital Blanchardstown, Dublin, Ireland; Johns Hopkins University, Baltimore, Maryland, United States; Icahn School of Medicine at Mount Sinai, New York, New York, United States; University of California, San Francisco, San Francisco, California, United States; Johns Hopkins University, Baltimore, Maryland, United States

## Abstract

Homebound older adults have high rates of multimorbidity, frailty, and functional decline, yet their care needs remain inadequately addressed. Using data from a scoping review we will identify and discuss care gaps in this vulnerable and growing population. The review followed the PRISMA-ScR framework, conducting systematic searches in PubMed, Embase, Web of Science, and CINAHL. A total of 159 studies met inclusion criteria. Two independent reviewers screened titles and abstracts, with a third resolving discrepancies. Six researchers extracted data using a standardized tool, capturing study characteristics, population details, and key findings. Themes were identified through consensus, resulting in four overarching categories. Studies highlight a need to address unmet care needs in dental care, nutrition, and fall prevention among the homebound. Over 90% of populations studied required dental treatment, yet fewer than 10% received care. Malnutrition was widespread, increasing frailty and hospitalization risk, yet nutritional interventions remain underutilized. Despite high fall rates, evidence on prevention strategies remains limited. Cognitive impairment and mental health conditions, particularly depression, were highly prevalent, yet access to neuropsychology services remained limited. Frailty and functional dependence were pervasive, contributing to high healthcare utilization, including emergency department visits and hospitalizations. Care gaps in advance care planning, social support, and rehabilitation were identified as barriers to improving quality of life. During the symposium, we will discuss how these gaps highlight an urgent need to transform care for homebound older adults. Expanding home-based medical care is essential to improving outcomes, reducing hospitalizations, and ensuring equitable, person-centered care.

